# Self-Control and the Effects of Movie Alcohol Portrayals on Immediate Alcohol Consumption in Male College Students

**DOI:** 10.3389/fpsyt.2014.00187

**Published:** 2015-02-03

**Authors:** Renske Koordeman, Doeschka J. Anschutz, Rutger C. M. E. Engels

**Affiliations:** ^1^Behavioural Science Institute, Radboud University, Nijmegen, Netherlands

**Keywords:** self-control, alcohol, movies, media, self-regulation

## Abstract

**Background:** In movies, alcohol-related cues are frequently depicted and there is evidence for a link between movie alcohol cues and immediate alcohol consumption. Less is known about factors influencing immediate effects movie alcohol exposure on drinking. The exertion of self-control is thought to be important in avoiding or resisting certain temptations.

**Aims:** The aim of the present study was to assess the immediate effects of movie alcohol portrayals on drinking of male social drinkers and to assess the moderating role of self-control in this relation. It was hypothesized that participants would drink more when exposed to movie alcohol portrayals and that especially participants with low self-control would be affected by these portrayals.

**Methods:** A between-subjects design comparing two movie conditions (alcohol or no portrayal of alcohol) was used, in which 154 pairs of male friends (ages 18–30) watched a 1-h movie in a semi-naturalistic living room setting. Their alcohol consumption while watching was examined. Participants completed a questionnaire assessing self-control as well as their self-reported weekly alcohol use. A multivariate regression analysis was conducted to test the effects of movie condition on alcohol comsumption.

**Results:** Self-control moderated the relation between movie condition and alcohol consumption. Assignment to the alcohol movie condition increased alcohol consumption during the movie for males with high self-control but not for males with low self-control.

**Conclusion:** Viewing a movie with alcohol portrayals can lead to higher alcohol consumption in a specific sample of young men while watching a movie.

## Introduction

Problematic alcohol use among young people is still a public health concern leading to many adverse consequences for young people, ranging from poor academic performances to physical and psychological health problems (1, 2). Exposure to alcohol-related cues can be considered as an important motivator of continued alcohol use as cues in the environment can activate cognitive processes such as goal activation and craving that relates to actual behavior (3–5). In movies, alcohol-related cues are frequently depicted (6, 7) and might trigger people to consume alcohol.

Cue-reactivity processes can be proposed in explaining increased drinking after exposure to alcohol cues. Biases in selective attention play an important role in the development and maintenance of addictive behaviors (8). It has been theorized by Robinson and Berridge (9, 10) that through classical conditioning, alcohol-related cues are able to produce a dopamine response. As a result, alcohol-related cues “grab” the attention and become highly motivationally salient and elicit approach behavior (9). The tendency among drinkers to automatically detect environmental alcohol cues may contribute to craving and drinking alcohol (11–13). The existence of selective attention to alcohol cues has been established among social and dependent drinkers [see review in Ref. (8)].

Recent studies demonstrated that the presence of alcohol cues in a movie increased the immediate alcohol consumption of young male students. Engels et al. (14) compared a movie with a high number of alcohol portrayals with a movie with only few alcohol portrayals and allowed participants to drink during the movie. Using an improved paradigm with a more advanced manipulation of the alcohol portrayals, Koordeman et al. (15) replicated these results. Two versions of the same movie were created that only differed in whether alcohol was displayed or not. It was found that men drank twice as much when there was alcohol in the movie than when there was no alcohol in the movie. Thus, these studies provided first evidence for a link between movie alcohol cues and immediate drinking. Less is known about factors influencing immediate effects of movie alcohol exposure on drinking.

A factor that might moderate people’s alcohol consumption in response to the sight of seeing actors’ drinking is self-control. The exertion of self-control is thought to be important in avoiding or resisting certain temptations. Self-control can be considered as a deliberate, conscious, effortful subset of self-regulation (16). A distinction can be made between state self-control and trait self-control (17). State self-control changes across situations and time and is thought to be affected by mood (18), previous attempts at self-control (19), motivation (20), and working memory capacity (21). Trait self-control is thought to be more stable over time and less susceptible to situational factors. People with high trait self-control are better able to control their impulses and to resist temptations. In the context of the present study, we focused on the role of trait self-control in the relation between movie alcohol exposure and alcohol use. Several studies showed that people with low self-control are more at risk of developing problematic drinking patterns (17, 22–24) and are less able to regulate their alcohol use (25, 26). Being exposed to alcohol cues (e.g., smell or sight) often provokes a desire to drink alcohol but may also trigger the conflicting intent not to drink (27) and this way causing a response conflict (25, 26). In such a situation, self-control might be required to resist the temptation to drink (28, 29). This fits with dual-process models, which assign a moderating role to a slower reflective and controlling system in addition to a more automatic system of cue reactivity (30). From this view, the automatic cue-reactivity processes will guide alcohol use, unless people have the ability and motivation to regulate this behavior. Thus, it might be more difficult for people with low self-control to resist alcohol use in response to movie alcohol cues than it is for people with high self-control.

The aim of the present study is to assess the immediate effects of movie alcohol portrayals on drinking of male social drinkers, and the role of self-control in this respect. We used a similar design as the Koordeman et al. (15) study in which we compared two versions of the same movie, one containing alcohol cues and the other containing no alcohol cues. Only men were selected since no effects of movie alcohol cues on drinking in females were found in Koordeman et al. (15). In addition, previous research comparing drinking behavior of men and women demonstrated that men were more inclined to imitate the sips of their drinking partner than women (31). When confronted with movie alcohol cues, men might generally be more susceptible to these cues since they are more often heavy drinkers (32, 33). We hypothesized that participants would drink more in the alcohol movie condition than participants in the non-alcohol condition. Furthermore, we expected that self-control would moderate the relation between movie alcohol exposure and alcohol consumption in a way that participants with low self-control would drink more in the alcohol condition compared to the non-alcohol condition, whereas we did not expect to find this difference (or less strong) in participants with high self-control.

## Materials and Methods

### Participants

In total, the sample consisted of 154 male university students aged 18–30 (*M* = 21.4, *SD* = 2.57) who watched a movie in dyads. Participants were recruited via flyers and an Internet system at Radboud University Nijmegen. In this system, students can voluntarily sign up for participation in ongoing research; however, students enrolled in courses of study (e.g., psychology) are obliged to participate in free to select experiments for a specific number of hours during their first year. All were asked to come with a friend. Students received either US$20 or course credits. The Institutional Review Board of the Faculty of Social Sciences of the Radboud University approved the study protocol and participants submitted written informed consents (15).

### Design

The study used a between-subject design with two experimental conditions in which participants were exposed to a 60-min version of the movie Stoller (34). Two versions of the movie were created; one showing scenes containing alcohol portrayal in addition to non-alcoholic scenes and the other showing exclusively non-alcoholic scenes. The alcohol and non-alcohol movie versions were carefully edited in such a way that they did not differ in length and storyline. The same scenes in both conditions were used as often as possible [cf. (15)]. Alcohol portrayal was defined as real or implied use of alcohol, including occasions where an alcoholic beverage was clearly in the possession of a character or was mentioned verbally. Alcoholic beverages that were displayed, but not implied as being consumed by a character were not coded as alcohol portrayal (35). In the alcohol movie condition, alcohol was portrayed for 490 s. After 10 min, alcohol was depicted for the first time. During the movie, the two main characters sipped alcohol 17 times. The movie *Get Him to the Greek* was chosen for its numerous scenes containing alcohol and for its appeal for male participants. The movie is about the unpredictable rock star Aldous Snow (Russel Brand) and his new manager Aron Green (Jonah Hill) who has 2 days to drag the unco-operative Aldous from London to Hollywood for a comeback concert.

### Procedure

Participants watched the movie in dyads in a room at the university designed to resemble a living room setting. To imitate natural TV-viewing conditions as closely as possible, the room was decorated with couches and decorations and a large TV-screen (15, 36). Sessions were conducted Tuesday to Friday from 5 until 9 p.m. and lasted 1.5 h. First, participants completed a questionnaire containing demographic questions and questions about their understanding of English to distract them from the real aim of the study. Next, they were told that they would watch a movie for 1 h. As a cover story, participants learned we were interested in their understanding of English movies and that they either watched a movie with Dutch subtitles or a movie without these subtitles and that we would ask questions about the movie afterward (in fact they all saw a movie without subtitles to not distract them from the portrayal). We told them we aimed at studying this in a home setting, and therefore, they could get free drinks and nuts from the refrigerator. Participants could choose from beer (Heineken, 5% alcohol), soda, or water in bottles of 20 or 25 cl.

During the experiment, video recordings were made by a hidden camera. The researcher coded the drinking behavior in a separate observation room. Participants were debriefed after the data collection was completed. We explicitly asked whether they agreed on using these observations, and none of them declined. After watching the movie, participants answered a second questionnaire containing questions about the movie, self-control, and their drinking behavior. Further, we asked them whether they were aware of the aim of the study. Of the participants, 17 indicated they had more or less understood the aim of the study. When conducting the analysis with and without these participants, the findings did not change (neither a substantial change in beta weights, nor in significance levels), so we decided to include them in the final sample. Participants who drank more than two alcoholic drinks were offered a taxi.

### Measures

After watching the movie, participants answered the following questionnaires. This might have affected the self-control measure.

#### Alcohol consumption (observations)

The researcher coded the amount of bottles and the amount of centiliters consumed and subtracted what was left in the bottle after the session ended (37).

#### Drinking habits

Weekly alcohol use was assessed with the questions: “Which of the past 7 days did you consume alcohol and how many glasses did you drank?” The sum total of the last 7 days was the measure used in the analysis (38). Problem drinking (39) was measured with six questions containing response possibilities of “yes” or “no.” For example: “In the past 12 months, have you tried to stop drinking without succeeding?” Further, binge drinking was measured with one question asking participants how many times they had drunk more than 6 alcoholic drinks per occasion over the past 12 months on a seven-point scale (40).

#### Evaluation of the movie

Six statements about the movie were presented, for which participants could indicate on a five-point Likert scale whether they agreed with the following statements (ranging from “totally do not agree” to “totally agree”). For example: “I liked the movie” (14). Cronbach’s α was 0.78.

#### Self-control

To assess self-control, a short and Dutch version of the self-control scale developed by Tangney et al. (17) was used, which aims to assess people’s ability to control impulses, emotions, thoughts, and performances (41). This version showed adequate reliability (42). Self-control was measured by 11 questions with five-point-Likert-scale response options ranging from 1 = *not at all* to 5 = *very much* (41). Examples of items are: “I have a hard time breaking bad habits” and “I wish I had more self-discipline.” Cronbach’s α was 0.74.

### Strategy for analyses

Means and standard deviations of all variables are given, split by condition. Possible differences between conditions on self-reported drinking measures, age, whether they had seen the movie before, evaluation of the movie, and day and time of the week the experiment took place where tested using a one-way ANOVA. Correlations between all model variables were calculated in order to examine the general relations between these variables and stable variables such as weekly drinking and problem drinking to assess which variables should be included as covariates in the main analyses.

To examine the effect of movie condition on alcohol consumption, we applied linear regression analysis using the software package MPLUS 5.1 (43), which allows the handling of nested data. Drinking of individuals is nested within dyads and failing to take this into consideration might lead to an inflation of effects. To correct for the potential non-independence of the data, the TYPE = COMPLEX procedure was used. This procedure corrects the standard errors of the parameter estimates for dependency leading to unbiased estimates (15, 44).

In a first model, we tested whether there were main effects of movie condition on alcohol consumption. In a second model, we examined whether self-control was a moderator between movie condition and alcohol consumption. Since the chi-square goodness-of-fit test is sensitive to sample size, the fit indices χ*^2^*, CFI (Comparative Fit Index), and RMSEA (Root-Mean-Square Error of Approximation) were utilized (45). In an additional analysis, it was tested whether there were differences between low and high self-control participants within the two movie conditions. For this analysis, a median split was conducted to construct a dichotomized self-control variable.

## Results

### Randomization

Randomization checks showed there were no differences between the two movie conditions concerning age, whether they had seen the movie before, evaluation of the movie, self-reported weekly alcohol use, binge drinking, and problem drinking (*p* > 0.05), indicating that randomization was successful.

### Descriptives

Table 1 shows the means and standard deviations of the model variables for the total sample and for the different movie conditions separately. On average, participants consumed 17.36 (*SD* = 14.26) alcoholic beverages per week. Of the participants, 72.3% reported drinking six or more glasses in one occasion at least once a month, and 15.2% of the sample reported problem drinking. Of all participants, 7.9% reported no drinking in the past week. Of the problem drinkers, 69.6% scored low on self-control. While watching the movie, 56 participants (36.1%) did not drink alcohol. In the alcohol condition 27 participants (34.2%) and in the non-alcohol movie condition 29 participants (38.2%) did not drink. The intraclass correlation (ICC) for drinking within the dyads was 0.67. Further, 13.2% reported that they had seen the movie before. Participants in the alcohol and non-alcohol movie condition equally liked the movie [*F*(1, 154) = 2.58, *p* > 0.05].

**Table 1:** **Sample means and SD**.

	Total	Alcohol condition	Non-alcohol condition	Range
Alcohol consumption^a^	30.74 (27.36)	31.96 (27.71)	29.47 (27.10)	0–150
Weekly alcohol consumption^b^	17.36 (14.26)	17.37 (14.23)	17.35 (14.38)	0+
Problem drinking	1.25 (1.22)	1.26 (1.22)	1.25 (1.23)	1–6
Binge drinking	4.96 (1.39)	5.08 (1.29)	4.86 (1.40)	1–7
Attitude toward movie	3.89 (3.07)	3.96 (0.51)	3.81 (0.64)	1–5

*^a^In centiliters*.

*^b^In consumptions per week*.

### Correlations

Pearson’s correlations showed that self-reported weekly consumption correlated with alcohol consumption while watching [*r* (286) = 0.46, *p* < 0.001] and was therefore added as covariate in subsequent analyses (see Table 2).

**Table 2:** **Pearson’s correlations (*N* = 154) between all model variables**.

	1	2	3	4
Weekly drinking	–			
Alcohol consumption lab	0.46**	–		
Problem drinking	0.31**	0.13	–	
Self-control	−0.30**	−0.21*	0.40**	–

*p < 0.01. **p < 0.001.

### Effects of movie alcohol portrayal on alcohol consumption

Two linear regression models were tested. The first model tested whether there was a main effect of movie condition on alcohol consumption. We found no effect of movie condition on alcohol consumption (β = −0.04, *p* = 0.71). A main effect of weekly drinking was found (β = 0.34, *p* < 0.001) implying that high weekly drinkers also consumed more alcohol in the lab.

**Figure 1:**
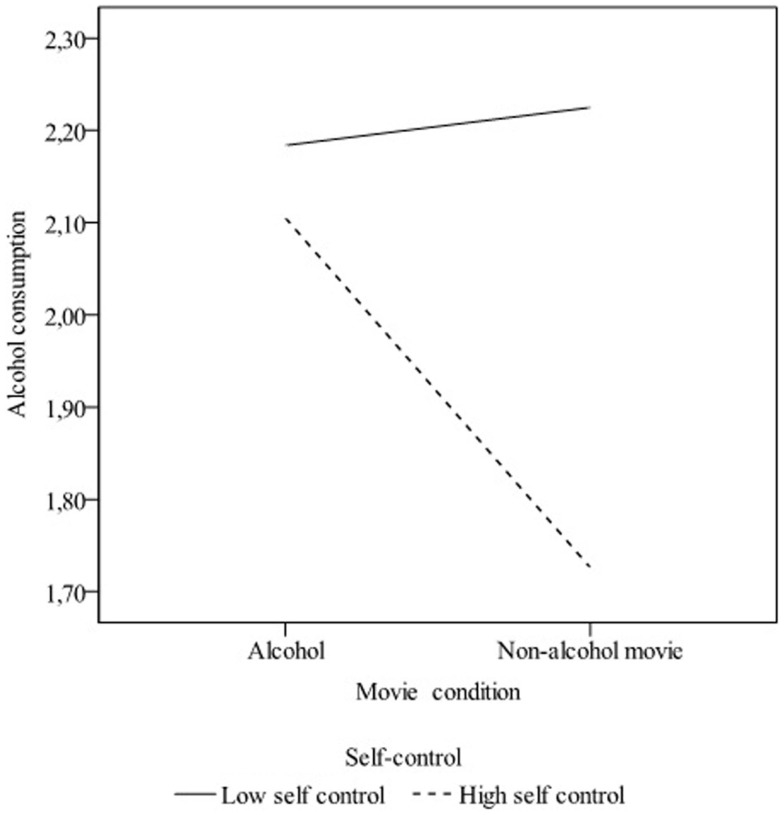
**Interaction between self-control and movie condition on alcohol consumption (in glasses of 15 cl)**.

In a second model, we tested the interaction effect of movie condition and self-control on alcohol consumption. The effect of movie condition on alcohol consumption just reached significance (β = 0.910, *p* = 0.05). A main effect of weekly drinking was found (β = 0.33, *p* < 0.001). Further, no main effect of self-control was found (β = 0.34, *p* = 0.15). However, there was a significant interaction between movie condition and self-control (β = −1.07, *p* = 0.03). Participants with a high score on self-control consumed more alcohol in the alcohol movie condition compared to the non-alcohol movie condition, whereas no differences between conditions were found for participants who score low on self-control (see Figure 1).

Additional analysis was conducted to compare low and high self-control participants within the movie conditions. It was found that within the non-alcohol condition low self-control participants consumed more alcohol than high self-control participants (β = −0.219, *p* = 0.034), whereas no differences between low and high self-control participants were found within the alcohol movie condition (*p* > 0.05).

## Discussion

The present study aimed to examine whether movie alcohol portrayals affected drinking behavior and whether this effect was influenced by trait self-control. The results did not confirm previous findings that male social drinkers consume more alcohol when being exposed to a movie with alcohol cues compared to a movie without any alcohol cues (14, 15). Yet, it was found that self-control moderated the effects of alcohol portrayal on drinking. In contrast to the hypothesis, only participants with high self-control drank more in the alcohol movie condition compared to the non-alcohol movie condition, whereas no differences between conditions were found for participants with low self-control.

In line with the extensive literature on the effects of self-control on alcohol use [e.g., Ref. (17, 22, 23, 28)], it was hypothesized that movie alcohol cues would affect drinking especially for men with low self-control. In both conditions, participants with low self-control consumed more alcohol than high self-control participants. Yet, only men with high self-control appeared to be affected by the movie alcohol portrayals. It seems that men with low self-control drank because there was an opportunity to drink regardless of whether there were alcohol cues on-screen. It has been proposed that individuals lower in self-control are less able to regulate their alcohol use and are more likely to violate self-imposed drinking limits (25, 26). It could be that the setting of the study in which alcohol was freely available and easily accessible, already prompted drinking in low-self-control people. A fridge with alcoholic and non-alcoholic beverages was placed in the room and the experimenter explained that the participants could drink whatever they liked. This might have been an enough reason for low self-control participants to drink. Another explanation could be that the non-alcohol movie clip contained elements of drugs and smoking. The alcohol and non-alcohol version of the movie were carefully edited to create versions that only differed in alcohol portrayals. Nevertheless, the non-alcohol movie still might have breathed the atmosphere of party and, therefore, could have (implicitly) stimulated participants with low self-control to consume alcohol as well. Moreover, participants’ weekly drinking correlated negatively with self-control. This might imply that men with low self-control have stronger associations with alcohol and partying, which could have caused little difference in drinking between conditions for this group. So, either both conditions stimulated them to drink, or explicit movie alcohol cues (in addition to a lab setting with alcohol) were not necessarily needed to encourage participants with low self-control to drink. It could be that participants with low self-control drank more at their own “pace,” independent of the actors’ drinking. In a similar vein, a study of Stoolmiller et al. (46) demonstrated that movie alcohol exposure was associated with greater increases of initiation of alcohol use among low sensation than among high sensation seeking adolescents and that their behavior was probably driven by other factors than media exposure.

However, high self-control people were affected by the alcohol cues in the movie. This is in line with a recent study, which showed that high self-control people when they are tempted are just as likely to lose self-control as low self-control people (47, 48). A meta-analysis by de Ridder et al. (49) suggested that trait self-control may operate more by way of establishing effective habits and routines than by resisting single temptations. So people with high trait self-control in general may avoid temptation rather than resisting it and therefore have less motivational conflict compared to low trait self-control people (47, 49). It could be that the lab setting with alcoholic beverages did not immediately trigger them to drink. Yet, when being exposed to movie alcohol cues in this lab setting, they were affected by these cues and inspired to drink. In contrast, people with low self-control are probably less able to avoid situations where they would be tempted; therefore, they have probably more self-control failures than high self-control people (e.g., take already a drink when being in a room with alcohol regardless of the movie cues). This could have contributed to higher average drinking levels of low self-control participants in both conditions.

Several limitations should be mentioned. First, participants filled out questions regarding self-control and alcohol-related behavior after the movie viewing which might have influenced their answers. Alcohol use could have decreased self-awareness and subsequently caused an overestimation of one’s self-control (50, 51). We chose to administer the questionnaires afterward to avoid participants knowing the real aim of the study. Moreover, administration prior to the experiment could have affected drinking during the experiment. Future studies could administer alcohol-related questions along with other types of lifestyle questions to disguise the true purpose of the study. In addition, a more context-relevant measure such as the Impaired Control Scale (52) that measures one’s perceived control over their alcohol use could be used in addition to the trait self-control scale. Second, we corrected statistically for the clustering of the data (i.e., drinking within dyads). Previous studies indicated that the majority of participants watch movies together with friends (15, 53). Still, watching the movie clip in dyads could have affected the drinking behavior and it is, therefore, important to replicate the findings with individual participants in the future. Furthermore, to assess the effect of peer drinking, future studies could examine the interplay between social/peer and movie cues by using a design in which confederates are instructed to sip and take drinks at specific moments.

We did not find differences in drinking between the alcohol and non-alcohol movie condition among participants as a whole. Perhaps, not all movie alcohol portrayals provoke the same effect on alcohol use. The movie used in the current study contained both positive (e.g., party, romance) and negative (alcohol addiction, drunkenness) portrayals of alcohol, whereas the movies used in previous studies mainly displayed positive alcohol portrayals (14, 15). Research showed that people had a more positive attitude about the effects of alcohol when they were exposed to alcohol portrayals with positive consequences than to portrayal with negative consequences (54, 55). In addition, de Graaf (56) demonstrated that people who were exposed to portrayal of negative consequences of alcohol use had more negative beliefs and attitudes toward alcohol use. In futures studies, it would be interesting to use designs in which specific social-emotional context of alcohol use can be variated to examine whether different movie alcohol portrayals provoke different responses on alcohol use.

To conclude, the current study showed that exposure to movie alcohol portrayals increased alcohol consumption while watching for people with high self-control but not for people with low self-control. These findings extend the findings of our previous experimental studies that alcohol use in movies encourages a specific sample of young males to drink (14, 15). Young people have substantial exposure to alcohol use in movies (6, 7). Reduce the number of movie alcohol cues, in order to reduce alcohol intake, may help. First, replication of the findings in different populations and other countries is necessary before strong policy recommendations can be made.

## Conflict of Interest Statement

The authors declare that the research was conducted in the absence of any commercial or financial relationships that could be construed as a potential conflict of interest.
